# Genetic Control of Canine Leishmaniasis: Genome-Wide Association Study and Genomic Selection Analysis

**DOI:** 10.1371/journal.pone.0035349

**Published:** 2012-04-25

**Authors:** Javier Quilez, Verónica Martínez, John A. Woolliams, Armand Sanchez, Ricardo Pong-Wong, Lorna J. Kennedy, Rupert J. Quinnell, William E. R. Ollier, Xavier Roura, Lluís Ferrer, Laura Altet, Olga Francino

**Affiliations:** 1 Departament de Genètica Animal, Centre de Recerca en Agrigenòmica (CRAG), Universitat Autònoma de Barcelona, Barcelona, Spain; 2 Departament de Ciència Animal i dels Aliments, Facultat de Veterinària, Universitat Autònoma de Barcelona, Barcelona, Spain; 3 Servei Veterinari de Genètica Molecular, Departament de Ciència Animal i dels Aliments, Facultat de Veterinària, Universitat Autònoma de Barcelona, Barcelona, Spain; 4 The Roslin Institute and R(D)SVS, University of Edinburgh, Easter Bush, Scotland, United Kingdom; 5 Centre for Integrated Genomic Medical Research (CIGMR), University of Manchester, Stopford Building, Oxford Road, Manchester, United Kingdom; 6 Institute of Integrative and Comparative Biology, University of Leeds, Leeds, United Kingdom; 7 Hospital Clínic Veterinari, Universitat Autònoma de Barcelona, Barcelona, Spain; Ohio State University Medical Center, United States of America

## Abstract

**Background:**

The current disease model for leishmaniasis suggests that only a proportion of infected individuals develop clinical disease, while others are asymptomatically infected due to immune control of infection. The factors that determine whether individuals progress to clinical disease following *Leishmania* infection are unclear, although previous studies suggest a role for host genetics. Our hypothesis was that canine leishmaniasis is a complex disease with multiple loci responsible for the progression of the disease from *Leishmania* infection.

**Methodology/Principal Findings:**

Genome-wide association and genomic selection approaches were applied to a population-based case-control dataset of 219 dogs from a single breed (Boxer) genotyped for ∼170,000 SNPs. Firstly, we aimed to identify individual disease loci; secondly, we quantified the genetic component of the observed phenotypic variance; and thirdly, we tested whether genome-wide SNP data could accurately predict the disease.

**Conclusions/Significance:**

We estimated that a substantial proportion of the genome is affecting the trait and that its heritability could be as high as 60%. Using the genome-wide association approach, the strongest associations were on chromosomes 1, 4 and 20, although none of these were statistically significant at a genome-wide level and after correcting for genetic stratification and lifestyle. Amongst these associations, chromosome 4: 61.2–76.9 Mb maps to a locus that has previously been associated with host susceptibility to human and murine leishmaniasis, and genomic selection estimated markers in this region to have the greatest effect on the phenotype. We therefore propose these regions as candidates for replication studies. An important finding of this study was the significant predictive value from using the genomic information. We found that the phenotype could be predicted with an accuracy of ∼0.29 in new samples and that the affection status was correctly predicted in 60% of dogs, significantly higher than expected by chance, and with satisfactory sensitivity-specificity values (AUC = 0.63).

## Introduction

Leishmaniasis is a vector-borne disease affecting humans and animals, caused by parasitic species of the genera *Leishmania* and transmitted by the bite of phlebotomine sand flies. Around the Mediterranean basin, visceral (VL) and cutaneous (CL) human leishmaniasis as well as canine leishmaniasis (CanL) are caused by *Leishmania infantum*. The current disease model for leishmaniasis suggests that infected individuals may live without progression towards clinical disease manifestation probably due to immune control of the infection.

The factors that determine whether individuals progress to clinical disease following *Leishmania* infection are unclear, but previous studies suggest a large contribution of the host genetic background, as reviewed elsewhere [1], [2]. Studies in mice [1] provided early support for a strong genetic component to susceptibility to *Leishmania* infection. In humans, most epidemiological studies [3], [4], [5], [6], candidate gene studies [7], [8], [9], [10], [11], [12] and genome-wide approaches [7], [13], [14] have offered further support for genetic susceptibility to leishmaniasis, however they did not specifically dissect the genetic factors that cause progression of the disease following infection. Some studies have investigated genetic differences between healthy infected and symptomatic individuals, but most of these were either not aimed to identify candidate loci [15], [16] or targeted at few candidate genes [17], [10], [18]. Only Jeronimo *et al.* [19] have studied progression of leishmaniasis following infection using a genome-wide linkage approach in humans based on a few hundred microsatellite markers. In dogs, genetic susceptibility to progression of disease from *Leishmania* infection is supported by the fact that the percentage of infected dogs in endemic areas is as high as 60% [20] whereas rates of clinical CanL are much lower in these areas [21], [22]. Similarly to familial aggregation and ethnic differences of leishmaniasis prevalence seen in humans, dog breeds show variable susceptibility to CanL. Some breeds such as Boxer, German shepherd and Rottweiler [23], [24], [25] appear more predisposed to overt CanL. In contrast, the Ibizan hound, a dog breed believed to have been relatively isolated in an endemic area such as Ibiza (Balearic Islands, Spain), is reported to be resistant to CanL [26].

Understanding the genomic factors controlling progression to clinical disease in dogs is critical since the dog is the main natural reservoir of *Leishmania infantum* infection for humans, and CanL is a disease of great importance in veterinary medicine because of its severity in the dog. Despite the importance of leishmaniasis in dogs, there have been very few genetic studies of this species and these have focused on a few candidate genes [27], [25], [28], [29], [30], which have confirmed some genes previously found in mice and humans. There have been no previous genome-wide studies of genetic susceptibility to visceral leishmaniasis in the dog.

The dog has been previously proposed as a comparative animal genetic model for disease mapping. For complex diseases, a strategy with a first genome-wide scan genotyping tens of thousands of single-nucleotide polymorphisms (SNPs) for a few hundreds of dogs from one or few breeds has been suggested [31], [32], [33] based on calculations of statistical power. This approach has been based on simulation studies. For complex phenotypes, these simulation studies demonstrate that 100–300 cases and 100–300 controls provide adequate power to detect alleles conferring 2 to 5-fold multiplicative risk [33]. As a proof of principle, the efficacy of the proposed design has recently been demonstrated on several different studies [34], [35], [36], [37], [38], [39], [40], [41], [42], [43], [44], [45]. Moreover, Daetwyler and collaborators [46] showed that the predictive accuracy depends upon the genomic structure of the species, and this is favorable for canine studies because of its low effective population increases the power in genomic selection techniques [47].

The aim of this work was therefore to carry out a genome-wide study of the genetic contribution to the progression of clinical CanL from *Leishmania* infection. Our working hypothesis were: (i) that the observed phenotypic variance in the progression of leishmaniasis in infected dogs is partly explained by the genetics of the host; (ii) that CanL is a complex disease with multiple loci involved and an environmental component; and (iii) that genomic information may be used to predict the progression of the disease. We applied both genome-wide association study (GWAS) and genomic selection approaches to a population-based case-control dataset of 219 dogs from a single dog breed (Boxer) genotyped for ∼170,000 single-nucleotide polymorphisms (SNPs) in order to study host genetic susceptibility to progression of clinical leishmaniasis from *Leishmania* infection. Firstly, we tried to identify loci in the canine genome associated with the disease progression phenotype. Secondly, we investigated the genetic component of the observed phenotypic variance. Thirdly, we examined whether genome-wide SNP data could be used to predict accurately the phenotype.

## Results

### Genome-wide scan of loci affecting disease progression

A GWAS analysis testing markers individually was performed in order to find loci associated with the progression to clinical CanL from *Leishmania* infection, using a dataset of 115 healthy infected and 104 affected Boxer dogs. All dogs had genotypes for 126,607 SNPs distributed across the genome.

Three statistical models were applied by fitting additional covariates in order to correct for the two confounding effects considered (described in **Materials and Methods**). When no covariates were included (Model 1), the strongest associations were found on *Canis familiaris* chromosomes (CFA) 1:39,058,553 bp (P_raw_ = 1.0×10^−5^, P_genome_ = 0.21), CFA 4: 68,238,371 bp (P_raw_ = 1.1×10^−5^, P_genome_ = 0.22) and CFA 20: 30,132,329 bp (P_raw_ = 2.5×10^−5^, P_genome_ = 0.43) (**Figure S1** and **Table S1**). Although healthy infected and affected samples generally clustered together in the MDS plot (**Figure S2**), genetic stratification was observed in our cleaned dataset based on the genomic inflation factor (λ = 1.29), with C1 capturing twice the stratification captured by C2. The associations on CFA1 and 4 remained when confounding effects were accounted for although significance did not reach the genome-wide level (**Table S1**). Genetic stratification, corrected by fitting the two first dimensions from the multidimensional scaling analysis (C1 and C2), is likely to explain part of the initial association in Model 1, as P_raw_ values for the ten strongest associated SNPs on CFA 1 and 4 were an order of magnitude higher when stratification was accounted for (Model 2). Nevertheless, associations of C1 and C2 with each of these markers were not significant (data not shown). Inclusion of dog lifestyle as a confounding effect did not affect the significance of the markers. After correction for the confounder effects (Model 3) the inflation factor was reduced to λ = 1.17 and this was not reduced by adding three additional MDS dimensions which altogether captured an extra 5% of the genetic variance in the markers (**Table S2**).

We examined candidate loci previously reported to have associations with host response to *Leishmania* infection and susceptibility to leishmaniasis in *Homo sapiens* (49 loci) and *Mus musculus* (33 loci) to test in a systematic way if any of these loci showed a stronger association in our canine dataset. When possible, these were mapped to their orthologues in the dog genome, and this was successful for 78 loci (95%; **Dataset S1**). We selected SNPs in the GWAS data contained within these candidate loci and their flanking regions (±1 Mb) and assigned them to sets of non-overlapping candidate regions. This resulted in 4,751 SNPs in 37 sets with a median of 108 SNPs (**Dataset S1**). Sets were tested one at a time for association with the phenotype controlling for within-set linkage disequilibrium (LD) and multiple testing arising from the number of SNPs in the set as described elsewhere [48] (r^2^ = 0.80 and p = 0.05 were used). Three sets of SNPs on CFA 4, and one each on CFA 9 and 10 showed an empirical set-specific p-value (EMP1)<0.05 (**Dataset S1**). The same sets showed EMP1<0.01 when r^2^ = 0.10 and p = 0.01 were applied (see **Materials and Methods**). Although EMP1 does not account for the fact that multiple sets are tested, the sets on CFA 4 showed EMP1 values notably lower than for other sets (**Figure S3**). The sets on CFA 4 spanned the region 61.2–76.9 Mb which had previously showed the strongest associations in the initial GWAS (**Table S1**). All the sets contain loci associated with *Leishmania* infection, and the three sets on CFA 4 included several genes (*Il7r*, *Lifr*, *C6*, *C7* and *Csf1r*) that lie within a locus involved in lesion development in murine *Leishmania major* infection [49], [50], [51], [52].

Finally, the same dataset used in the GWAS was analysed using genomic selection with the BayesB method [47] with some modifications previously published [53]. Briefly, the BayesB method first proposed by Meuwissen *et al.* [47] is a Bayesian model in which the effect of SNPs on the total genetic values are predicted simultaneously, with an *a priori* assumption that only few SNPs are useful for predicting the trait. With the modified BayesB method we used (from now onwards referred just as BayesB for simplicity), Models 1–3 produced a similar genome-wide plot of both estimated marker effects (**Figure S4**) and the proportion of realisations a given marker was estimated to have a non-zero effect (data not shown), with a most detectable peak on CFA 4:61–77 Mb. This region overlapped with both the strongest association in GWAS and the region in which SNP sets covering candidate genes were significant (EMP1<0.01).

### Estimating genetic variance in the phenotype

GWAS methodology is concerned with identifying individual SNPs that may be a causative variant for the phenotype or in LD with such a variant. Despite the failure to detect any such SNP, it was possible to detect genetic variation relating to the leishmaniasis phenotype. Two different approaches were adopted, the first using a modified BayesB methodology [53] and the second a Restricted Maximum Likelihood (REML) methodology implemented within the GCTA package [54]. The estimates of heritability obtained were 0.64 and 0.58 (s.e. 0.17) from BayesB and GCTA, respectively (**Table 1**). These estimates were corrected for genetic stratification (C1, C2) and lifestyle. Note that these estimates are likely to be biased upwards because of the selection of the samples contributing to the study – as would be expected in a case-control study. Given the uncertainty of the actual prevalence of the disease we decided to explore this using GCTA by varying the prevalence from 0.01 to 0.6. As show in **Table S3**, in all cases heritability was found notably greater than zero and it went down to 0.32 with prevalence equal to 0.01.

**Table 1:** Summary results from the BayesB and GCTA analyses.

	Model 1	Model 2	Model 3	Model 4
***BayesB***				
Posterior 1–*π* (%)	1.65	1.57	1.54	1.57
*h^2^*	0.61	0.63	0.64	0.65
***GCTA***				
*h^2^* (s.e.)	0.53 (0.18)	0.55 (0.18)	0.58 (0.17)	0.59 (0.17)

The estimates for the percentage of markers affecting the phenotype (1–π) and its heritability (*h^2^*) are shown for the different statistical models: Model 1 included no covariates; Model 2 included the first two dimensions of the MDS analysis; Model 3 included the first two dimensions of the MDS analysis plus the lifestyle; Model 4 included an additional dimension of the MDS analysis to Model 3.

Using BayesB the fraction of markers contributing to the genetic variance was estimated as 0.015 (s.e. 0.011), however experience with such methods suggests that this fraction is sensitive to the distribution of allele effects that is assumed (results not shown). The inclusion of an additional MDS dimension (C3) did not change the results compared with Model 3.

### Prediction of the phenotype

Cross-validation was used to test the predictive potential of genomic evaluation. Five cross-validation sets (denoted A–E) were produced at random from the full dataset to estimate the predictive benefit when new individuals, which have not been used to estimate the effects of markers and covariates, are genotyped in order to predict their phenotypes. Two approaches to assess the predictive value were adopted: the accuracy to predict the phenotype and the capability to diagnose individuals from genomic information.

#### Accuracy

The correlation between predicted fitted values for the new individuals and their known actual phenotype was calculated as a measure of accuracy (*r*) for predicting the phenotype. The Model 1 results suggest that the combined SNP effects predict the phenotype with an accuracy of 0.18 and that, by comparison with Model 2, little accuracy is added by including covariates correcting for genetic stratification (**Table 2** and **Table 3**). Including lifestyle, which was identified as a risk factor in previous analyses, improved the accuracy to 0.29 (**Table 4**). Still, the key question is whether the genomic data adds accuracy and this was assessed in different ways.

**Table 2:** Summary of cross-validation results after constructing five sets (labelled A–E), showing the predictive accuracy when the set is excluded from the training set for Model 1.

Model 1						
Set	A	B	C	D	E	A–E
N_training_	175	175	177	176	173	
N_cases_	21	21	20	20	22	104
***Full model***						
Accuracy (*r*)	0.02	0.09	0.41	0.49	0.07	0.18
(95% CI)	(−0.28, 0.32)	(−0.21, 0.38)	(0.13, 0.64)	(0.22, 0.69)	(−0.23, 0.35)	(0.05, 0.30)
Empirical significance	0.42	0.34	<0.01	<0.01	0.44	<0.01
***Permuted genotypes***						
Accuracy (r)	−0.11	−0.05	−0.17	−0.23	−0.13	−0.14
(95% CI)	(−0.39, 0.19)	(−0.34, 0.25)	(−0.45, 0.14)	(−0.49, 0.08)	(−0.41, 0.16)	(−0.27, −0.01)

Empirical significance was obtained from the fraction of permutations that showed a correlation higher than in the real data.

**Table 3:** Summary of cross-validation results after constructing five sets (labelled A–E), showing the predictive accuracy when the set is excluded from the training set for Model 2.

Model 2						
Set	A	B	C	D	E	A–E
N_training_	175	175	177	176	173	
N_cases_	21	21	20	20	22	104
***Full model***						
Accuracy (*r*)	0.05	0.05	0.41	0.53	0.12	0.20
(95% CI)	(−0.26, 0.34)	(−0.25, 0.34)	(0.12, 0.64)	(0.27, 0.71)	(−0.18, 0.39)	(0.07, 0.32)
Empirical significance	0.37	0.27	0.02	0.03	0.34	0.04
***Permuted genotypes***						
Accuracy (r)	−0.03	−0.09	0.09	0.11	0.07	0.02
(95% CI)	(−0.32, 0.27)	(−0.38, 0.21)	(−0.22, 0.38)	(−0.20, 0.40)	(−0.23, 0.35)	(−0.11, 0.15)
***Covariates alone***						
Accuracy (r)	0.003	−0.06	0.23	0.43	0.17	0.11
(95% CI)	(−0.29, 0.30)	(−0.35, 0.24)	(−0.08, 0.50)	(0.15, 0.65)	(−0.13, 0.43)	(−0.02, 0.24)

Empirical significance was obtained from the fraction of permutations that showed a correlation higher than in the real data.

**Table 4:** Summary of cross-validation results after constructing five sets (labelled A–E), showing the predictive accuracy when the set is excluded from the training set for Model 3.

Model 3						
Set	A	B	C	D	E	A–E
N_training_	175	175	177	176	173	
N_cases_	21	21	20	20	22	104
***Full model***						
Accuracy (*r*)	0.10	0.14	0.46	0.56	0.23	0.29
(95% CI)	(−0.20, 0.38)	(−0.16, 0.42)	(0.18, 0.67)	(0.32, 0.74)	(−0.06, 0.49)	(0.16, 0.41)
Empirical significance	0.48	0.24	0.02	0.01	0.46	0.03
***Permuted genotypes***						
Accuracy (r)	0.09	−0.01	0.28	0.29	0.26	0.15
(95% CI)	(−0.22, 0.37)	(−0.31, 0.29)	(−0.03, 0.54)	(−0.01, 0.54)	(−0.03, 0.51)	(0.02, 0.28)
***Covariates alone***						
Accuracy (r)	0.11	0.03	0.35	0.43	0.34	0.22
(95% CI)	(−0.19, 0.40)	(−0.27, 0.32)	(0.05, 0.59)	(0.15, 0.65)	(0.05, 0.57)	(0.09, 0.35)

Empirical significance was obtained from the fraction of permutations that showed a correlation higher than in the real data.

Firstly, cross-validation was performed on permuted data prior to the running of the BayesB analyses, where genotypes were randomized with respect to both phenotypes and covariates, whilst the link between phenotypes and covariates was maintained. In general, accuracy values were notably lower with permuted data than with the actual data, regardless of which of Models 1 to 3 were fitted. Within-set accuracies from permuted data were very close to zero when no covariates (Model 1) and genetic stratification (Model 2) were included. Statistical significance was observed only when lifestyle was included (Model 3), which confirms the earlier result that lifestyle has predictive value.

Secondly, to test the contribution of the genomic data, the predictions obtained from the BayesB analysis were decomposed into the component from the covariates and the component from the SNPs. The SNP component was then permuted within the cross-validation set as described in the Materials and Methods, but maintaining the link between the predictor from covariates and the phenotypes. For each permutation the accuracy of prediction was calculated. **Tables 2** **,** **3** **,** **4** show that the accuracy from the observed data with the true link between phenotypes and genotypes was in the upper tail of the distribution of accuracies (P<0.05). Collectively this demonstrates the Models have significant predictive value and that, within the predictor, the genomic data makes a significant contribution to the accuracy.

Finally, the magnitude of the benefit from the genomic data was assessed by predictions that excluded all genomic data. Overall accuracy obtained by C1 and C2 as explanatory factors alone was not significant (**Table 3**). Accuracy using only covariates in Model 3 was significant although the accuracy achieved was only half the value obtained from the full Model (**Table 4**). Altogether, these three ways to look at the data proved that prediction of the phenotype was more accurate when genetic markers were included.

Nevertheless, as may be expected from the relatively small data sets, there is considerable variation among the cross validation sets, and confidence intervals within individual cross-validation sets are large. Predictive accuracies were significant in sets C and D, but were not significant in sets A, B and E (**Table 2**), coinciding with a slightly higher posterior fraction of markers with a non-zero (1–π) effect for sets A, B and E than for C and D (data not shown). Overall, there was an improvement in prediction by using SNPs.

#### Prediction of the trait

**Figure 1:**
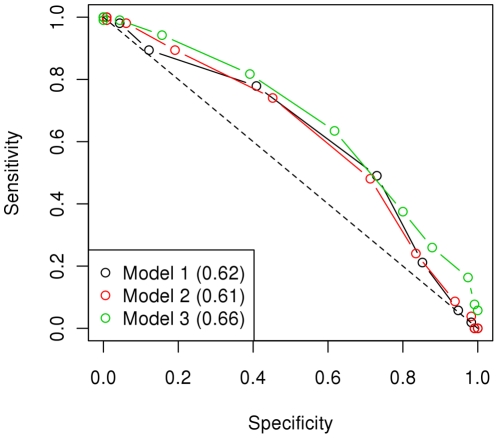
Receiver Operating Characteristic (ROC) curves. Sensitivity and specificity values were obtained for increasing classification thresholds to produce the ROC curves. In the legend, the values for the area under the ROC curve (AUC) are indicated in parenthesis for each model. AUC can range between 0.5 (randomness, dashed line) and 1.0 (ideally).

**Figure 2:**
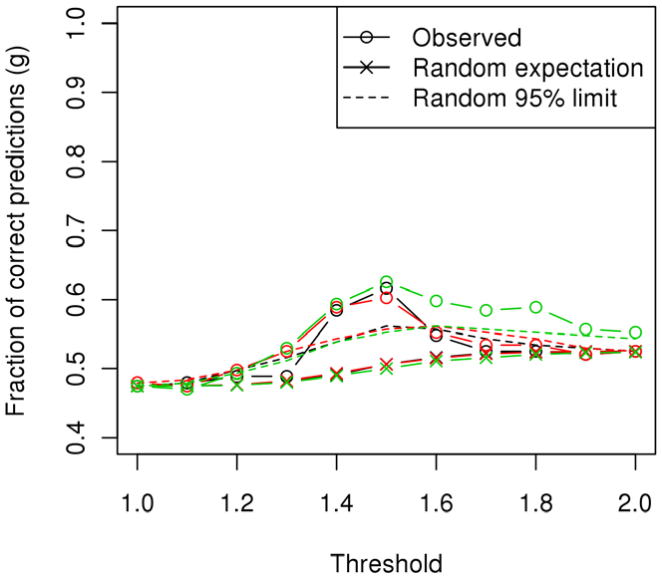
Fraction of correct predictions. For increasing classification thresholds percentages of correct classifications were compared to those expected by chance. Calculations for the random expectation and the random 95% limit were drawn from a hypergeometric distribution and are detailed in **Text S1**.

Our second approach to assess the capability of our data to be used for prognosis of disease development required individuals to be classified as either healthy infected or affected for increasing thresholds of fitted values. Note that the phenotype was defined as one or two for healthy infected and affected, respectively, and therefore fitted values were approximately in this range. Receiver operating characteristic (ROC) curves were generated from sensitivity and specificity values for different thresholds and the area under the curve (AUC) was calculated as an indicative of the balance between sensitivity and specificity. AUC values were notably higher than randomness and Model 3 achieved the best performance (**Figure 1**). Regardless of the model, a threshold of 1.5 to diagnose individuals would reach the highest fraction of correct predictions (*g*), notably higher than the expected by chance alone (for Model 3, *g*_1.5_ = 0.63; *g*_95%limit_ = 0.55) (**Figure 2**).

## Discussion

In this study we have explored the contribution of genetic loci in the dog genome for determining clinical progression of disease following *Leishmania* infection and how such information may be used to predict disease course. Our first analysis was focused on identifying individual loci in the canine genome which contributed medium to large effects for determining disease development. Different analyses associated CFA 4: 61–77 Mb. The strongest association in the GWAS analysis was for markers in this region, even when we considered confounding factors such as lifestyle and genetic stratification, whose causes are discussed below. However, these associations were not significant when corrected for multiple testing (**Figure S1**, **Table S1**). The lack of genome-wide significance at the individual SNP level may indicate that our study was underpowered for GWAS due to the small sample size of our study. However the size of the study was at the lower end of the range of 100–300 cases and 100–300 controls that has been suggested for GWAS in dogs in complex diseases [33]. The lack of genome-wide significance may also be evidence of a complex genetic nature for leishmaniasis. This provides justification for the genomic selection approach which is more suited to prediction of complex traits (e.g. [47]).

Interestingly, when we tested for association focusing only on SNPs residing within candidate loci related to host response to *Leishmania* and susceptibility to leishmaniasis in humans and mice [50], [55], [56], loci on chromosome 4: 61–77 Mb were significant after correcting for multiple testing and linkage disequilibrium (**Dataset S1** and **Figure S3**).

In addition, from the BayesB analysis, markers in this region of CFA 4 had a larger estimated effect on the phenotype than other genome-wide markers (**Figure S4**). Chromosome 4: 61–77 Mb is syntenic to a locus that mediates host response to *Leishmania major* in mice, which includes the candidate genes *Il7r*, *Lifr*, *C6* and *C7* [50]. *Il7r* (CFA 4: 75.8 Mb) is of special interest as, although healthy infected and affected samples showed similar MAF and observed heterozygosity values along CFA 4: 61–77 Mb, in both groups three SNPs (CFA 4: 75.7–75.9 Mb) flanking *Il7r* significantly deviated from HWE (p-value<10^−5^) (data not shown). Extended patterns of markers deviating from HWE may indicate copy number variants. Variation in the number of copies between affected and healthy infected cannot be detected through differences in genotype frequencies though it might affect the phenotype. In fact, structural variations have been described for the syntenic region in the human genome [57], [55], [58], which encompasses *SPEF2*, *CAPSL*, *UGT3A1* and *UGT3A2* in addition to *IL7R*. In mice, structural variation has also been reported for a shorter region overlapping *Ugt3a1* [59]. However, replication in an independent sample is needed to confirm the association on chromosome 4, as well as those on chromosomes 1 and 20, and the identification of these regions only represents a first discovery step for a better understanding of the genetic variants that control genetic susceptibility to clinical progression of leishmaniasis from *Leishmania* infection.

Next, we studied the extent to which the additive effects of loci throughout the genome determine the disease development following *Leishmania* infection. Our data suggest that the trait is complex with many different gene segments contributing to the phenotype and that the genetic variance may explain as much as 60% of the total observed phenotypic variance. Whilst this estimate was fairly consistent across the different methodologies used for its estimation (**Table 1**), the estimation is made more complex and very likely to be biased upward, by the case-control nature of the data. This is the first clear evidence that there is a significant genetic component to leishmaniasis in dogs within breeds. In addition, it is the first heritability estimate for progression of clinical leishmaniasis from *Leishmania* infection in any species, although an estimate of heritability for a marker of healed *Leishmania* infection and protection against subsequent reinfection in humans has been reported [19].

An important finding of this study was that whilst no single SNP was found to be reliably predictive, there was significant predictive value of the genomic data through using the genomic evaluation as proposed by Meuwissen *et al.* [47]. The best predictor included information on lifestyle as well as the genomic predictor, but it was clearly established that the genomics made a substantial contribution to the accuracy. The model including the covariates and the genomic data reached an accuracy of 0.29 for a dog that was outside the current dataset (e.g. a newborn dog), and thus is only weakly predictive of the phenotype. However three points should be remembered. Firstly this accuracy was achieved using 80% of the data (the other 20% were used for cross-validation), and that the total data consisted of only 219 infected animals, of which only 104 had developed the disease. Secondly, this accuracy is the prediction of a phenotype and not the underlying genetic liability, and the accuracy of predicting the genetic liability is likely to be greater. In random sample with continuous traits the accuracy would be scaled by 1/h (>1) where h is the square root of the heritability. The structure of the data prevents us from proposing any correction. Thirdly the value of using genomics is that the genomic data can be accumulated over time with increasing accuracy of prediction. One might anticipate that further collection of cases and controls would increase accuracy to levels that have the potential for making a clinical impact on breeding for resistance away from the development of pathology, i.e. toleration of the parasite.

Finally, we would like to comment on the possible causes of the genetic stratification seen in our dataset, which especially affected the GWAS results and could only be reduced to λ = 1.17, and to compare with other GWAS in dogs. Roughly half of the dozen published GWAS in dogs provided information with regard to stratification. Three studies [37], [45], [43] observed good clustering of cases and controls when plotting the first two MDS dimensions, in spite of different geographical origin of the samples in the study from Madsen et al.. Barber et al. [42] also used MDS in order to detect stratification and excluded a good number of outlier samples. Wilbe et al. [34] and Downs et al. [60] reported inflation factor values, before correction, of 1.3 and 1.4, respectively. Both studies observed clustering of either samples with similar geographic provenance [34] or known to be related [60] and performed a Cochran-Mantel-Haenszel stratified analysis within the clusters as a measure of correction. However, no value of the inflation factor after the correction was presented. Only Olsson and collegues reported an inflation factor of 1.2 after removing two outliers in the MDS plot [35]. We consider that it is unlikely that our lambda value was inflated due to population stratification because we neither observed geographical clustering of samples within Spain (the majority of the samples were collected from different areas in the country) nor differentiation of samples collected in other countries (i.e. Italy, Greece and Portugal). It is reasonable to think that geographical stratification would have been noticed if present, as it has happened with some other canine GWAS. Although population or geographical stratification is a common cause of increased inflation factor, there are other confounder effects that can produce the same results [61]. We tried to avoid differential bias by following the same procedures in the collection of samples and clustering of samples that went through different DNA extraction protocols or genotyping batches was ruled out. Although we tried to avoid family structure by not including members of the same family, cryptic relatedness might have certainly inflated the lambda value. Nonetheless, we note that lambda values >1.05 are typically considered to denote stratification in human studies [61]. Although this is a statistical rule-of-thumb and it should be the same regardless of the species, we wonder if certain relatedness owing to founder effects, inbreeding, popular sire effects and repeated mating might be inherent to GWAS in dogs in spite of a careful study design.

## Materials and Methods

### Ethics statement

The dogs in the study were examined during routinary veterinary procedures by the veterinary clinics participating in the study. All samples were collected for routine diagnostic and clinical purposes. The samples were obtained during veterinary procedures that would have been carried out anyway and DNA was extracted from residual surplus of samples and used in the study with verbal owner consent. This is a very special situation in veterinary medicine. As the data are from client-owned dogs that underwent normal veterinary exams, there was no “animal experiment” according to the legal definitions in Spain and the United Kingdom, and approval by an ethical committee was not necessary.

### Study population and epidemiology

The study population consisted of a single breed of dogs (Boxer). This design was chosen as the use of a single breed for the first stage of GWAS in dogs will increase power by reducing effects of genetic differentiation between breeds and increasing the degree of linkage disequilibrium [33]. Moreover, this breed appears more predisposed to overt CanL than others [23], [62], [63] . An age criterion for study inclusion was applied (see below) as it has been reported that age distribution of the prevalence of infection follows a bimodal pattern, with the first peak including dogs diagnosed at 2 to 4 years of age and the second peak including dogs about 7 years old [64]. The study was carried out in collaboration with the Hospital Clínic Veterinari of the Universitat Autònoma de Barcelona (HCV-UAB) and therefore most Boxers included were from the metropolitan area of Barcelona, Spain, where the HCV-UAB is located. A number of Boxers from other areas where the disease is endemic were also included (Spain, Greece, Italy and Portugal).

### Veterinary clinics recruitment and samples collection

Veterinary clinics and dog owners were encouraged to participate in the study, in the case of the latter through their veterinary centre. Two millilitres of EDTA peripheral blood and 2 ml of serum were required. In addition, other tissues (*e.g.* bone marrow) as well as conjunctiva or lesion swabs were received occasionally. With regard to CanL, no pre-screening of the samples sent to the laboratory was done by the veterinary clinics. Dogs affected by CanL included those with a documented history of disease, undergoing a relapse or newly diagnosed. For those showing a disease episode when samples were collected, additional samples were requested one month after treatment or if requested by the referring clinician in order to both confirm the diagnosis and help inform then clinician on treatment response. For healthy infected dogs (see below), additional samples and medical information were requested to confirm the absence of CanL development. Inclusion of additional samples relied on the collaboration of veterinary clinics.

### Phenotype definition, clinical classification and laboratory tests

Dogs were classified by clinical signs, clinical biochemistry, direct parasite detection and anti-*Leishmania* immune reactions into the following groups: (i) healthy infected: healthy and >4 years old but with evidence of prior infection and (ii) affected: manifest clinical disease and diagnosed before the age of 4 years. Ages were recorded as age-of-onset of disease for affected and current age at sample collection for healthy infected dogs. Lifestyle (*i.e.* living indoors, outdoors, both or undetermined), gender, level of relatedness and geographic location of origin were also collected. *Leishmania* quantitative polymerase chain reaction (qPCR) and anti-*Leishmania* Enzyme-Linked ImmunoSorbent Assay (ELISA) were performed on all samples from all dogs, although additional results from direct parasite detection and anti-*Leishmania* immune tests were provided by veterinary clinics for most samples. *Leishmania* qPCR was performed at the Servei Veterinari de Genètica Molecular, UAB, as described previously [62] and ELISA was performed at UNIVET Servicio de Diagnóstico Veterinario SL, UAB, as described elsewhere [63].

### DNA extraction, SNP genotyping and data quality control

DNA was extracted from peripheral blood and bone marrow samples using either QIAamp® DNA Blood Mini Kit (QIAGEN) or by conventional phenol-chloroform DNA extraction and deproteinization methods. All samples were genotyped using the Illumina CanineHD BeadChip (174,376 markers) [65] at the Centre National de Génotypage, France. Data cleaning was conducted using PLINK [48] and R version 2.13.0 [66] packages. Quality control was performed independently on two genotyping batches. In total eight samples with call rate <90% were excluded. Intensity probes were excluded together with markers on the boundary autosomal region on the CFA X and SNPs on the non-pseudoautosomal region on CFA X for which heterozygous genotypes in male samples were observed. Markers with call rate <90% were also excluded. Multidimensional scaling (MDS) analysis based on the genotypes was performed to detect samples with a very different genetic content (explained below). Three affected dogs were excluded because they appeared as outliers when the first two dimensions from the MDS analysis were plot (data not shown). After data cleaning 115 healthy infected and 104 affected dogs remained. In addition, markers were filtered to have a minor allele frequency (MAF) >1.5% and a Hardy-Weinberg Equilibrium (HWE) test p-value>0.005 (a threshold set based on the empirical distribution of our data). This left 126,607 markers for analysis. Finally, for logistic regression and BayesB analyses, one SNP of a pair was removed for those SNP pairs showing complete genotypic correlation, resulting in 99,997 SNPs left for analysis.

### Statistical analysis

#### Covariates

Two confounding effects were considered and fitted into the statistical analyses: genetic stratification and dog lifestyle. Using PLINK [48], an identical-by-state correlation matrix for *n* individuals was calculated from which *n* dimensions were extracted using MDS analysis, resulting in a matrix of *n*-samples by *n*-dimensions eigenvalues. The fraction of genetic variance explained by each dimension was calculated as the variance for a given dimension along all samples divided by the sum of variances for all the dimensions extracted. The eigenvalues for the first two dimensions (C1 and C2) of the MDS analysis were used as continuous covariates. For simplicity, only C1 and C2 were used because the fraction of additional genetic variance explained by each of the subsequent 217 MDS dimensions extracted was minimal (**Figure S2**). Although healthy infected and affected samples generally clustered together in the MDS plot (**Figure S2**), genetic stratification was observed in our cleaned dataset based on the genomic inflation factor (λ = 1.29), with C1 capturing twice the stratification captured by C2. Therefore, C1 and C2 values for each sample were fitted as continuous covariates in the indicated models. CanL is known to be a complex disease with an environmental component and thus dogs living outdoors, more exposed to infection, are believed to more frequently develop the disease. Hence, lifestyle was also included as a factor in the analyses for the models indicated. For Model 3, the inflation factor was reduced to λ = 1.17. In the logistic regression, lifestyle was fitted as a factor (one degree of freedom) using dummy variables for indoors, indoors/outdoors, outdoors and ‘undetermined’. In the genomic selection analyses, lifestyle was considered as a categorical covariate with four levels. Three genetic models varying in whether covariates were fitted were defined to explain the phenotype (y), treated as binary (i.e. either healthy infected or affected):

Model 1: y∼SNPs

Model 2: y∼SNPs+C1+C2

Model 3: y∼SNPs+C1+C2+lifestyle

### *GWAS and candidate genes analysis*

Markers were tested for association using the Cochran-Armitage for trend test (Model 1) and logistic regression (Models 2 and 3). Genome-wide significance (P_genome_) was obtained after 10,000 permutations. Based on the permutations carried out in our dataset, the uncorrected p-value that would reach genome-wide significance (at the 5% level) after correction for multiple testing in our study would be p = 2.08×10^−6^. Formally, first, for each permutation the maximum statistic across all SNPs was recorded and, second, from this distribution of maximum statistics, the statistic in the top 5% is used to give the p = 2.08×10^−6^ that would be significant after permutations.

Candidate loci reported as related to host response to *Leishmania* and susceptibility to leishmaniasis in *Mus musculus* and *Homo sapiens* [56], [67] were used to retrieve homologous loci in *C. familiaris* using Biomart [68] with CanFam 2.0. Sets were defined with SNPs in the Illumina's CanineHD Beadchip residing within the retrieved candidate loci and their flanking regions (±1 Mb). Loci for which at least one SNP overlapped were merged into the same set (**Dataset 1**). Set-based association tests were performed as described in PLINK [53] with two different sets of parameters: (1) r^2^ = 0.80, p-value = 0.05; and (2) r^2^ = 0.10, p-value = 0.01. In both cases, a maximum set size of 10 SNPs (∼10% of the median set size) was used. The Cochran-Armitage for trend test was used and 10,000 within-set permutations were conducted to obtain empirical set-based p-values (EMP1).

#### Modified BayesB method

Datasets were analysed with the BayesB method [47] with some modifications previously published [53] assuming Models 1–3. The phenotype was treated as continuous. A flat prior distribution for the proportion of markers with non-zero additive effect (1–π) was set to follow a beta distribution with parameters α = 1 and β = 1 and a starting value of 0.2. An informative distribution for the variance for the additive parameter was set to follow an inverse chi-squared distribution with two degrees of freedom (*ν* = 2) and a scale parameter (*S*) of 0.001 (weak prior). The Markov chain Monte Carlo (MCMC) of BayesB was run for 160,000 cycles and the first 10,000 cycles were discarded as burn in; 3,000 realisations of sampling were performed with 50 cycles between realisations. Absolute variation between each of the 3,000 sampled values of posterior *S* and their prior *S* (either *S* = 0.001 or *S* = 0.1) was calculated and a Welch two sample t-test was applied. The same test was applied to the sampled values of (1–π) produced by each of weak and stronger *S* priors. BayesB produced estimates of the genomic breeding values (GEBV) and of the effects of C1, C2 and each lifestyle category, which were used to calculate fitted values of the phenotype (ŷ) according to the different predictive models (**Text S1**).

In order to assess the effect of the weak informative prior distribution used for the variance of the additive parameter on the resulting posterior value, for Model 1 the analysis was repeated with *S* = 0.1 (strong prior), which was 100 times higher than the value used otherwise. In absolute values, the posterior *S* value changed a 58.7% respect to the weak prior (*S* = 0.001) whereas this variation was significantly greater, 78.9%, for *S* = 0.1 (p-value = 7×10^−6^). Another effect of giving a stronger prior *S* was that the posterior proportion of markers with a non-zero effect (1–π) was 100 times lower compared to the obtained for the weak prior (0.02% and 1.65%, respectively, p-value = 2×10^−16^) and the fraction of genetic variance was also lower (0.27 and 0.61, respectively). Moreover, the genome-wide pattern of estimated SNP effects was notably different depending on the prior given. With a weak prior most markers had non-zero but very low estimated effect (10%-quantile = 10^−5^) whereas a small fraction of SNPs (10%-quantile = 0) had 10-fold estimated effects with a stronger prior. Altogether these results can be explained by a scenario in which fewer SNPs with greater effect contribute to the phenotype when a greater prior variance of the additive parameter is allowed.

#### Restricted Maximum Likelihood (REML) analysis (GCTA software)

When calculating the genetic relationship matrix (GRM) with GCTA [54], no adjustment was specified to correct for imperfect LD between genotyped markers and causal loci. REML analyses were run assuming Models 1–3. As input parameters, genetic and environmental variances were not specified and default values of 0.12 for both were used. Model 3 was run with varying phenotype prevalence values from 0.01 to 0.60 in order to explore the sensitivity of estimates to prevalence.

#### Cross-validation

Samples were assigned randomly to one of five training sets (denoted A–E) so that (i) each training set had a size of approximately 4/5 of the full unpermuted dataset and (ii) in each training set the proportions of samples belonging to each phenotype (either affected or healthy infected) and lifestyle categories were approximately as in the full dataset. Samples not included in each training set were used as testing data. For each training set, BayesB was run to both estimate E_C1_, E_C2_, E_life_ from samples in the training set and produce GEBV for the testing data samples and then calculate their fitted values, according to the corresponding predictive model.

Accuracy (*r*) was calculated as the correlation between fitted values (ŷ) and true phenotypes (y). In each testing set, *r* was calculated as a measure of accuracy to predict the phenotype. The overall correlation for the full unpermuted dataset was calculated by combining the predictions across sets. The contribution of markers to the accuracy was analysed in three ways. First, GEBV generated with BayesB were permuted before the calculation of fitted values. In this way, the correspondence between phenotypes and covariates was not altered. Within each model, 100 sets of permuted GEBV, resulting in 100 sets of permuted fitted values, were generated for each set. The empirical p-value for the real data was computed as the fraction of permuted sets with a lower p-value that the real data. Second, genotypes were randomized respective to phenotypes and covariates, which were kept as in the original data. The BayesB analysis was then run and cross-validation applied as explained before. Third, fitted values were calculated using uniquely covariates, i.e. GEBV were not used.

For Models 1–3, receiver operating characteristic (ROC) curves were calculated as follows. A fitted value threshold was set so that below or above it individuals were predicted to be healthy infected or affected, respectively. Specificity, sensitivity and fraction of correct predictions (*g*) values were calculated (**Text S1**) for increasing thresholds of fitted values and ROC curves were generated by plotting specificity against sensitivity. The area under the ROC curve (AUC) was calculated as a measure of similarity between specificity and sensitivity.

## Supporting Information

Figure S1 **Single-marker genome-wide association plot for Model 1 after 10,000 permutations with the strongest associations indicated.**
(TIFF)

Figure S2 **Genetic stratification.** (**A**) relative genetic variance explained by the 219 MDS dimensions extracted; (**B**) MDS plot for the first two MDS dimensions (C1 and C2) with healthy infected and affected samples coloured differently. The percentage of relative genetic variance explained by each dimension is indicated as well as the genomic inflation factor (lambda).
(TIFF)

Figure S3 **Distribution of EMP1 across SNP sets of candidate regions.** Sets comprise SNPs in the following regions: 6 (CFA 4:61.2–63.2 Mb), 7 (CFA 4: 70.5–74.5 Mb), 8 (CFA 4: 74.8–76.9 Mb), 19 (CFA 9: 40.0–46.5 Mb) and 22 (CFA 10: 29.6–31.5 Mb).
(TIFF)

Figure S4 **Genome-wide plot of the absolute mean SNP effects estimated with BayesB for Model 1 (A), Model 2 (B) and Model 3 (C).** The peak on CFA 4: 61–77 Mb (red segment) consistent across Models 1–3 coincided with both the strongest association in GWAS analysis and the region in which SNP sets covering candidate genes were significant (EMP1<0.01).
(TIFF)

Table S1 **Strongest associations from each region identified in the GWAS analysis.** BICF2P1345879 was not used in models 2 and 3 because, for logistic regression, SNPs were pruned based on LD (see **Materials and Methods**).The closest marker, <6 Kb upstream, was BICF2P813758 at 20:30,126,633 bp (Model 2: P_raw_ = 4.4×10^−4^, P_genome_>0.50, OR = 0.33; Model 3: P_raw_ = 6.2×10^−4^, P_genome_>0.50, OR = 0.34). Choice of SNPs representing each genomic region was based on the strongest associations in Model 1. *Canis familiaris* genes (CanFam_2.0) were retrieved using Biomart and associated gene names are given, with the exception of some for which no gene name was available and the Ensembl ID is given instead. The same information is presented for the strongest associations on chromosomes 9 and 10 from the set-based analysis.
(XLS)

Table S2 **Genomic inflation (λ) was not affected by fitting additional MDS dimensions as covariates of the model.**
(DOC)

Table S3 **Sensitivity of heritability (** ***h^2^*** **) estimation using GCTA to prevalence of the phenotype is shown for Model 3.**
(DOC)

Text S1 **Fitted values, fraction of correct predictions, sensitivity and specificity calculation.**
(DOC)

Dataset S1 **Candidate genes analysis: (A) candidate genes and loci described in** ***H. sapiens*** **and** ***M. musculus*** **and retrieved genomic positions in** ***C. familiaris*** **; (B) sets of non-overlapping candidate regions plus their ±1 Mb-flanking regions; (C) results from the set-based association study.**
(XLS)
