# Supplementary figures and images for: Genetic Control of Canine Leishmaniasis: Genome-Wide Association Study and Genomic Selection Analysis
Source: PLoS One. 2012 Apr 25;7(4):e35349. doi: 10.1371/journal.pone.0035349 (PMC3338836; doi:10.1371/journal.pone.0035349)

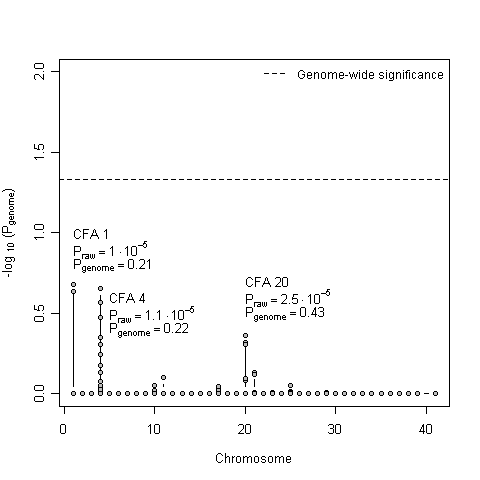

Supplement: Figure S1 — Single-marker genome-wide association plot for Model 1 after 10,000 permutations with the strongest associations indicated. (TIFF) [file pone.0035349.s001.tif]

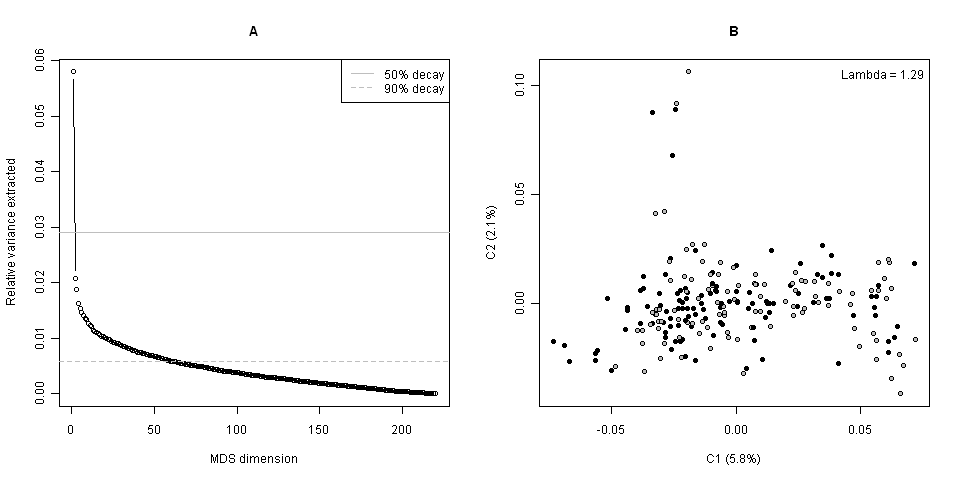

Supplement: Figure S2 — Genetic stratification. (A) relative genetic variance explained by the 219 MDS dimensions extracted; (B) MDS plot for the first two MDS dimensions (C1 and C2) with healthy infected and affected samples coloured differently. The percentage of relative genetic variance explained by each dimension is indicated as well as the genomic inflation factor (lambda). (TIFF) [file pone.0035349.s002.tif]

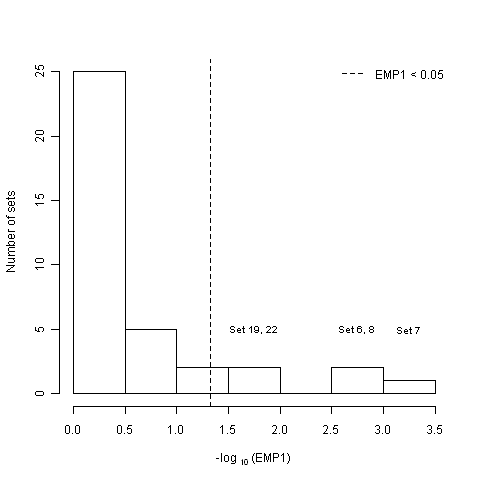

Supplement: Figure S3 — Distribution of EMP1 across SNP sets of candidate regions. Sets comprise SNPs in the following regions: 6 (CFA 4:61.2–63.2 Mb), 7 (CFA 4: 70.5–74.5 Mb), 8 (CFA 4: 74.8–76.9 Mb), 19 (CFA 9: 40.0–46.5 Mb) and 22 (CFA 10: 29.6–31.5 Mb). (TIFF) [file pone.0035349.s003.tif]

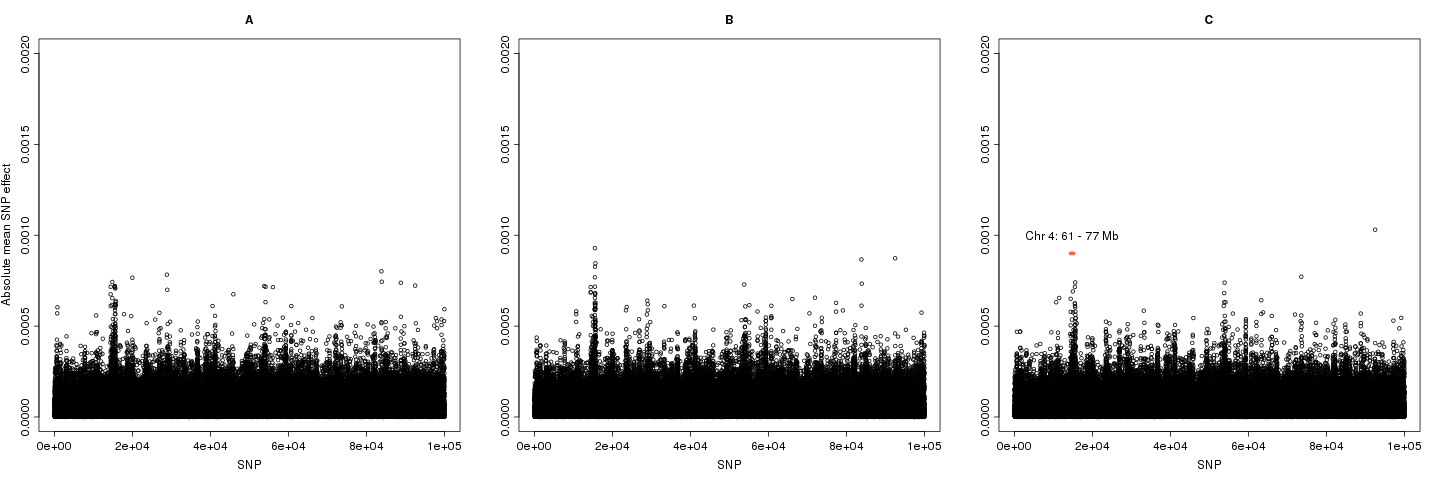

Supplement: Figure S4 — Genome-wide plot of the absolute mean SNP effects estimated with BayesB for Model 1 (A), Model 2 (B) and Model 3 (C). The peak on CFA 4: 61–77 Mb (red segment) consistent across Models 1–3 coincided with both the strongest association in GWAS analysis and the region in which SNP sets covering candidate genes were significant (EMP1<0.01). (TIFF) [file pone.0035349.s004.tif]
