# Supplementary material for: Genetic Control of Canine Leishmaniasis: Genome-Wide Association Study and Genomic Selection Analysis
Source: PLoS One. 2012 Apr 25;7(4):e35349. doi: 10.1371/journal.pone.0035349 (PMC3338836; doi:10.1371/journal.pone.0035349)
Supplement: Table S2 — Genomic inflation (λ) was not affected by fitting additional MDS dimensions as covariates of the model. (DOC) [file pone.0035349.s006.doc]

## Table S2

| MDS dimensions^a^ | Relative genetic variance (%)^b^ | λ | s.e. |
| --- | --- | --- | --- |
| C3 | 1.83 | 1.19 | 0.00018 |
| C3, C4 | 1.64 | 1.17 | 0.00017 |
| C3, C4, C5 | 1.52 | 1.19 | 0.00017 |

## ^a^In addition to MDS dimensions C1, C2 and lifestyle.

## ^b^It refers to the relative genetic variance explained by the last MDS dimension included.
