# Supplementary material for: Genetic Control of Canine Leishmaniasis: Genome-Wide Association Study and Genomic Selection Analysis
Source: PLoS One. 2012 Apr 25;7(4):e35349. doi: 10.1371/journal.pone.0035349 (PMC3338836; doi:10.1371/journal.pone.0035349)
Supplement: Table S3 — Sensitivity of heritability ( h2 ) estimation using GCTA to prevalence of the phenotype is shown for Model 3. (DOC) [file pone.0035349.s007.doc]

## Table S3

| **Prevalence** | ***h^2^*** | **s.e.** |
| --- | --- | --- |
| 0.01 | 0.32 | 0.10 |
| 0.05 | 0.49 | 0.15 |
| 0.10 | 0.61 | 0.18 |
| 0.20 | 0.76 | 0.23 |
| 0.30 | 0.85 | 0.25 |
| 0.40 | 0.90 | 0.27 |
| 0.50 | 0.91 | 0.27 |
| 0.60 | 0.90 | 0.27 |

## 
