# Supplementary material for: Genetic Control of Canine Leishmaniasis: Genome-Wide Association Study and Genomic Selection Analysis
Source: PLoS One. 2012 Apr 25;7(4):e35349. doi: 10.1371/journal.pone.0035349 (PMC3338836; doi:10.1371/journal.pone.0035349)
Supplement: Text S1 — Fitted values, fraction of correct predictions, sensitivity and specificity calculation. (DOC) [file pone.0035349.s008.doc]

## Text S1

**Calculation of Fitted values (ŷ)**

BayesB produced estimates of the genomic breeding values (GEBV) and of the effects of C1, C2 and each lifestyle category, which were used to calculate fitted values of the phenotype (ŷ) according to the different predictive models:

$$\begin{matrix} Model 1: \widehat{y}=\mu\cdot1_{n}+GEBV \\ \\ Model 2: \widehat{y}=\mu\cdot1_{n}+E_{C1}\cdot(C1-\overline{C1})+E_{C2}\cdot(C2-\overline{C2})+GEBV \\ \\ Model 3: \widehat{y}=\mu\cdot1_{n}+E_{C1}\cdot(C1-\overline{C1})+E_{C2}\cdot(C2-\overline{C2})+E_{life}\cdot X_{life}+GEBV \end{matrix}$$

Where, ŷ is the vector of fitted values, μ is the overall mean, 1_n_ is a vector of *n*-samples ones, E_C1_ and E_C2_ are the estimated effects for C1 and C2, respectively, C1 and C2 are vectors with the eigenvalues for C1 and C2 MDS dimensions, respectively, $\overline{C1}$and $\overline{C2}$are the mean values for C1 and C2, respectively, E_life_ is a vector with the estimated effect for each lifestyle, X_life_ is a matrix with as many rows as samples and as many columns as lifestyle levels with 1 degree of freedom, taking value 1 for the recorded lifestyle and zero otherwise. Throughout the analyses, correlations between actual phenotypes and fitted values were calculated as the Pearson product-moment correlation coefficient (*r*).

**Fraction of correct predictions (*g*)**

X was defined as a random variable following a hypergeometric distribution describing the number of healthy infected declared correctly, with parameters: a, number of true affected, c, number of true healthy infected, n = a + c, and r, sample of individuals declared at random to be healthy infected (r ≤ n). Thus:

$$\begin{matrix} E\left(X\right)=\frac{r\cdot c}{n} \\ \\ Var\left(X\right)=\frac{r\cdot c\left(n-r\right)\cdot\left(n-c\right)}{n^{2}\cdot\left(n-1\right)}=\frac{r\cdot a\cdot c\cdot\left(n-r\right)}{n^{2}\cdot\left(n-1\right)} \end{matrix}$$

Y was defined as the total correct predictions:

$$\begin{matrix} Y=X+a-\left(r-x\right)=X+a-r+X=2\cdot X+\left(a-r\right) \\ \\ E\left(Y\right)=E\left(2\cdot X+\left(a-r\right)\right)=E\left(2\cdot X\right)+E\left(a-r\right)=2\cdot E\left(X\right)+\left(a-r\right)=\frac{2\cdot r\cdot c}{n}+\left(a-r\right) \\ \\ Var\left(Y\right)=Var\left(2\cdot X+\left(a-r\right)\right)=Var\left(2\cdot X\right)=4\cdot Var\left(X\right)=\frac{4\cdot r\cdot a\cdot c\cdot\left(n-r\right)}{n^{2}\cdot\left(n-1\right)} \end{matrix}$$

Z was defined as the fraction of correct calls:

$$\begin{matrix} Z=\frac{Y}{n}=\frac{2\cdot X+\left(a-r\right)}{n} \\ \\ E\left(Z\right)=E\left(\frac{2\cdot X}{n}+\frac{a-r}{n}\right)=\frac{2}{n}\cdot E\left(X\right)+\frac{a-r}{n}=\frac{2\cdot r\cdot c}{n^{2}}+\frac{a-r}{n} \\ \\ Var\left(Z\right)=Var\left(\frac{2}{n}\cdot X+\frac{a-r}{n}\right)=\frac{4}{n^{2}}\cdot Var\left(X\right)=\frac{4\cdot r\cdot a\cdot c\cdot\left(n-r\right)}{n^{4}\cdot\left(n-1\right)} \end{matrix}$$

The random 95% limit of Z was calculated from X | F(X) ≥ 0.95, where F is the distribution function.

**Sensitivity and specificity**

The number of individuals correctly predicted as healthy infected (X_observed_) and correctly predicted as affected (n – r_observed_) was used to calculate:

$$\begin{matrix} Specificity=\frac{X_{observed}}{c} \\ \\ Sensitivity=\frac{\left(n-r_{observed}\right)}{a} \\ \\ g=\frac{2\cdot X_{observed}+\left(a-r_{observed}\right)}{n} \end{matrix}$$
